# Structure-Informed Prioritization of Phytochemical Antistreptococcal Candidates: Integrated Molecular Docking and In Vitro Proof-of-Concept Validation Against *Streptococcus agalactiae* and *Streptococcus pyogenes*

**DOI:** 10.3390/cimb48090906

**Published:** 2026-09-04

**Authors:** Momir Dunjic, Stefano Turini, Tatjana Novakovic, Lazar Nejkovic, Zhao Jing, Marija Dunjic, Katarina Dunjic

**Affiliations:** 1 Faculty of Medicine, University of Pristina, 38220 Kosovska Mitrovica, Serbia; novakovictanja65@gmail.com; 2 Faculty of Pharmacy, 21000 Novi Sad, Serbia; 3 Alma Mater Europaea (AMEU-ECM), 2000 Maribor, Slovenia; 4 Division of Environmental Medicine and Security, Capri Campus Forensic and Security, Anacapri, Capri Island, 80071 Naples, Italy; 5 Institute of Medical Research (IMI), University of Belgrade, 11029 Belgrade, Serbia; 6 School of Medicine, University of Belgrade, 11000 Belgrade, Serbia; lazar.nejkovic@gmail.com (L.N.); marijadunjic@yahoo.com (M.D.); dunjickatarina@gmail.com (K.D.); 7 Institute of Basic Research in Clinical Medicine, China Academy of Chinese Medical Sciences, Beijing 100700, China; hhzhaojing@hotmail.com

**Keywords:** molecular docking, *Streptococcus agalactiae*, *Streptococcus pyogenes*, Rex, protein tyrosine phosphatase, biofilm, phytochemicals, Spearman rank correlation, principal component analysis, in silico–in vitro concordance, localized antimicrobial formulation

## Abstract

Localized phytochemical formulations may provide complementary strategies for controlling mucosal colonization by *Streptococcus agalactiae* and *Streptococcus pyogenes*, but computational prioritization requires orthogonal biological validation. This study integrated molecular docking against the redox-sensing transcriptional repressor Rex of *S. agalactiae* and a protein tyrosine phosphatase target of *S. pyogenes* (SP-PTP) with in vitro broth microdilution, biofilm, target-bridging, and epithelial-tolerance experiments. Isoflavone showed the most favorable natural-compound interaction with Rex (ΔG = −8.3 kcal/mol), whereas secoisolariciresinol diglucoside (SDG) led the natural-ligand ranking for SP-PTP (ΔG = −5.2 kcal/mol). The complete formulation produced MIC50 values of 0.031% and 0.063% *v*/*v* against *S. agalactiae* and *S. pyogenes*, respectively; inhibited biofilm formation by 89.2% and 80.1%; and preserved >92% epithelial viability at 1× MIC. Among the three natural candidates with complete matched broth data, target-normalized docking and composite MIC50/MIC90/MBC50 ranks were perfectly concordant in both species; pooled species-stratified Spearman analysis yielded ρ = 1.000 (exact *p* = 0.0556; *n* = 6). In contrast, the extended panel including antibacterial comparators showed negligible concordance (ρ = 0.081; *p* = 0.7826; *n* = 14). Principal component analysis assigned 74.8% of variance to an in vitro potency axis and 24.6% to a largely orthogonal docking axis. Thus, the experiments confirmed the target-specific ordering of the natural candidates but did not support extrapolation of docking rank across mechanistically heterogeneous antibacterial classes. Intracellular target inhibition, genetic causality, and component synergy remain unestablished.

## 1. Introduction

*Streptococcus agalactiae* (group B Streptococcus, GBS) and *Streptococcus pyogenes* (group A Streptococcus, GAS) are clinically important Gram-positive organisms whose pathogenic repertoires encompass asymptomatic colonization, mucosal disease, and invasive infection [1,2,3,4]. Their capacity to form biofilms and coordinate virulence programs facilitates persistence under environmental and host-derived stress [5,6,7,8,9]. In the female genital tract, species-directed local interventions are, therefore, of interest as adjuncts to established diagnostic, antimicrobial-stewardship, and prevention strategies.

The emergence and dissemination of antimicrobial resistance require therapeutic-development pipelines that separate preliminary prioritization from biological proof. Molecular surveillance, standardized resistance definitions, and burden estimates have clarified the scale of this challenge [10,11,12,13,14,15]. Local delivery may limit systemic exposure, but it also introduces specific requirements: adequate contact time, activity against biofilm-associated cells, physicochemical stability, and preservation of epithelial integrity.

Molecular docking is useful for ranking ligand–target compatibility, provided that docking energy is not equated with whole-cell efficacy [16,17,18]. Natural products and essential-oil constituents offer chemically diverse scaffolds with reported antimicrobial or antibiofilm activity [19,20,21,22,23,24,25,26]. Terpinen-4-ol has membrane-active and antibiofilm properties [21]; SDG provides a lignan scaffold with broad biological relevance [22,23]; and isoflavones such as biochanin A exhibit pleiotropic pharmacology [27]. Formulation-level performance may also benefit from multi-component physicochemical interactions and advanced delivery systems [24].

Because docking energies and concentration-based biological endpoints differ in scale and may be related monotonically rather than linearly, cross-layer validation benefits from a rank-based framework. Spearman rank correlation quantifies monotonic concordance without imposing a linear model, whereas principal component analysis (PCA) summarizes the multivariate covariance structure and identifies dominant, potentially orthogonal response axes [28,29].

The present study was designed as a preclinical, species-resolved investigation. It evaluated a panel of phytochemicals and conventional comparators against two regulatory targets: Rex, a redox-sensitive regulator implicated in *S. agalactiae* adaptation and virulence [30], and an SP-PTP target selected for *S. pyogenes*. Docking-derived ranks were compared with experimentally measured antimicrobial, antibiofilm, target-bridging, and epithelial-tolerance endpoints. The objective was to test whether the in vitro experiments confirmed the target-specific hierarchy identified in silico and to quantify the strength and scope of this agreement by Spearman correlation and PCA, without inferring clinical efficacy.

## 2. Materials and Methods

### 2.1. Study Design and Experimental Workflow

The study followed a sequential computational-to-preclinical framework. Ligands were first ranked by predicted binding energy against the species-specific target. The same candidates, a complete oil-based formulation, vehicle controls, and conventional antibacterial comparators were then evaluated by broth microdilution, biofilm, recombinant-protein, and epithelial-cell assays. Docking scores were treated exclusively as prioritization indices; no causal mechanism was inferred from ΔG alone.

Following computational prioritization, laboratory experiments were conducted to evaluate antimicrobial activity, biofilm inhibition and disruption, target-level responses, and epithelial tolerance. The experimental dataset was generated using the procedures described below, with vehicle, untreated, positive-irritant, and antibacterial controls applied as appropriate. Experimental results were analyzed independently of the docking energies and were subsequently compared with the in silico rankings to assess cross-layer concordance.

### 2.2. Ligands, Bacterial Targets, and Formulation

The candidate set comprised terpinen-4-ol, γ-terpinene, 1,8-cineole, α-terpinene, α-terpineol, p-cymene, α-pinene, an isoflavone scaffold, and SDG. Penicillin G, ampicillin, cefazolin, clindamycin, and vancomycin were included as reference comparators. The *S. agalactiae* target was the redox-sensing transcriptional repressor Rex; the *S. pyogenes* target was a protein tyrosine phosphatase designated SP-PTP.

The complete test article was a defined oil-based formulation intended for local vaginal delivery. Analytical standards were obtained from Sigma-Aldrich/Merck (Darmstadt, Germany) or Cayman Chemical (Ann Arbor, MI, USA). Formulation composition was verified by GC–MS for volatile components and by HPLC-DAD or LC–MS/MS, as appropriate, for non-volatile phenolic and lignan constituents. Batch-specific composition and quality-control criteria were recorded during experimental testing.

### 2.3. Molecular Docking and Rank Interpretation

Docking was performed with the 1-Click Docking platform (Mcule, Palo Alto, CA, USA; provider version not specified) using the selected target structures and ligand inputs. For each target, the principal output was predicted binding free energy (ΔG, kcal/mol), with more negative values interpreted as more favorable ligand–target compatibility. Ligands were categorized as more favorable, intermediate, or lower priority within each target-specific distribution.

Docking ranks were interpreted at the species level. Rex-directed values were compared with GBS-associated biological outputs, whereas SP-PTP-directed values were compared with GAS-associated outputs. Differences in permeability, solubility, chemical stability, membrane activity, and formulation-mediated exposure were recognized as potential sources of docking-to-biology discordance [16,17,18].

### 2.4. Bacterial Strains, Authentication, and Biosafety

Clinical isolates of *S. agalactiae* and *S. pyogenes* were evaluated, with *S. agalactiae* ATCC 12386 and *S. pyogenes* ATCC 19615 included as quality-control comparators. Species authentication was performed by MALDI-TOF mass spectrometry, Lancefield grouping, and routine accredited laboratory verification before experimental testing.

All viable-organism procedures were performed by trained personnel in a certified BSL-2 laboratory using a Class II biological safety cabinet, institutionally approved SOPs, validated decontamination procedures, and the applicable CLSI/EUCAST-aligned workflow. Operational pathogen-handling parameters were governed by controlled institutional SOPs and are therefore not reproduced in detail.

### 2.5. Broth Microdilution and Bactericidal Endpoints

MIC and MBC values were determined by broth microdilution in supplemented cation-adjusted Mueller–Hinton broth. Test conditions included the complete formulation, individual purified constituents, vehicle controls, and antibacterial comparators. Hydrophobic candidates were evaluated with a solvent/emulsifier control confirmed to be microbiologically inert at the final assay concentration. Optical density and visual growth endpoints were recorded using a validated microplate reader workflow.

The recorded outputs included MIC range, MIC50, MIC90, MBC range, and MBC/MIC ratio. Because the complete formulation and purified compounds were expressed in different units, cross-condition comparisons were confined to normalized rank displays; absolute potency comparisons across % *v*/*v* and mg/L were not made.

### 2.6. Biofilm Inhibition and Disruption

Biofilm assays evaluated both prevention of biomass accumulation and disruption of established biofilm. Crystal violet biomass, resazurin-based metabolic activity, live/dead fluorescence, and confocal imaging, where applicable, were used as orthogonal readouts. Outcomes were expressed as percentage reduction relative to untreated and vehicle controls and summarized as mean ± SEM across experimental replicates.

### 2.7. Target-Bridging Assays

Recombinant *S. agalactiae* Rex and *S. pyogenes* SP-PTP were used as non-infectious mechanistic bridges. Rex thermal stabilization was assessed by differential scanning fluorimetry as the ligand-associated change in melting temperature (ΔTm). SP-PTP function was quantified as residual phosphatase activity after exposure to each ligand or the formulation. These assays tested target-level concordance rather than intracellular target occupancy.

### 2.8. Epithelial Viability and Barrier Tolerance

Local tolerance was evaluated in VK2/E6E7 vaginal epithelial cells and Ect1/E6E7 ectocervical epithelial cells (both obtained from ATCC, Manassas, VA, USA). CellTiter-Glo viability, lactate dehydrogenase release, and transepithelial electrical resistance (TEER) were used to measure metabolic viability, membrane injury, and barrier integrity. Vehicle and positive-irritant controls were included. Test concentrations were anchored to the experimentally determined microbiologically active range.

### 2.9. Statistical and Reporting Strategy

Continuous values were summarized as mean ± SEM. Rank-based interpretation was used because docking energies and biological responses occupy different scales and do not imply linear dependence. Target-specific docking ranks were compared directionally with antimicrobial, biofilm, target-bridging, and epithelial-tolerance endpoints. All values and labels were checked for internal consistency, and measured experimental outputs were evaluated separately from predicted docking energies.

### 2.10. Spearman Rank Correlation and Principal Component Analysis

Cross-layer concordance was computed on compound–species observations with a docking score and complete MIC50, MIC90, and MBC50 data. Within each species, docking energy was directionally transformed as −ΔG, and each concentration endpoint was transformed as −log10(concentration in mg/L), so that larger values consistently denoted more favorable affinity or potency. Average percentile ranks were assigned to ties, and the in vitro composite rank was the arithmetic mean of the MIC50, MIC90, and MBC50 ranks. The right-censored *S. pyogenes* terpinen-4-ol MBC50 (>259 mg/L) was retained as censored and assigned the least-favorable rank; it was not treated as an exact concentration. The complete formulation was excluded because no single-ligand docking score exists, and cefazolin was excluded because matched broth endpoints were unavailable. No missing values were imputed.

The prespecified candidate-focused analysis comprised isoflavone, SDG, and terpinen-4-ol, the three natural candidates with complete paired data. For this analysis, docking and composite potency ranks were re-expressed as percentiles within the three-candidate set for each species before pooling the six compound–species observations. Spearman’s rank coefficient (ρ) was accompanied by a two-sided exact *p* value obtained by exhaustive within-species permutation (3! × 3! = 36 assignments). An extended analysis included the three candidates and four antibacterial comparators (penicillin G, ampicillin, clindamycin, and vancomycin; *n* = 14), with species-specific and pooled coefficients and two-sided asymptotic *p* values. Biofilm and target-bridging coefficients were evaluated as endpoint-specific sensitivity analyses on the available matched subsets. PCA was performed on the standardized four-variable matrix of within-species docking, MIC50, MIC90, and MBC50 ranks for the extended panel. The first two component scores and variable loadings were displayed in a biplot. Given the small, partially repeated compound panel, all correlation statistics were interpreted as exploratory effect-size estimates rather than as confirmatory evidence.

## 3. Results

### 3.1. Target-Specific Docking Prioritization

**Figure 1:**
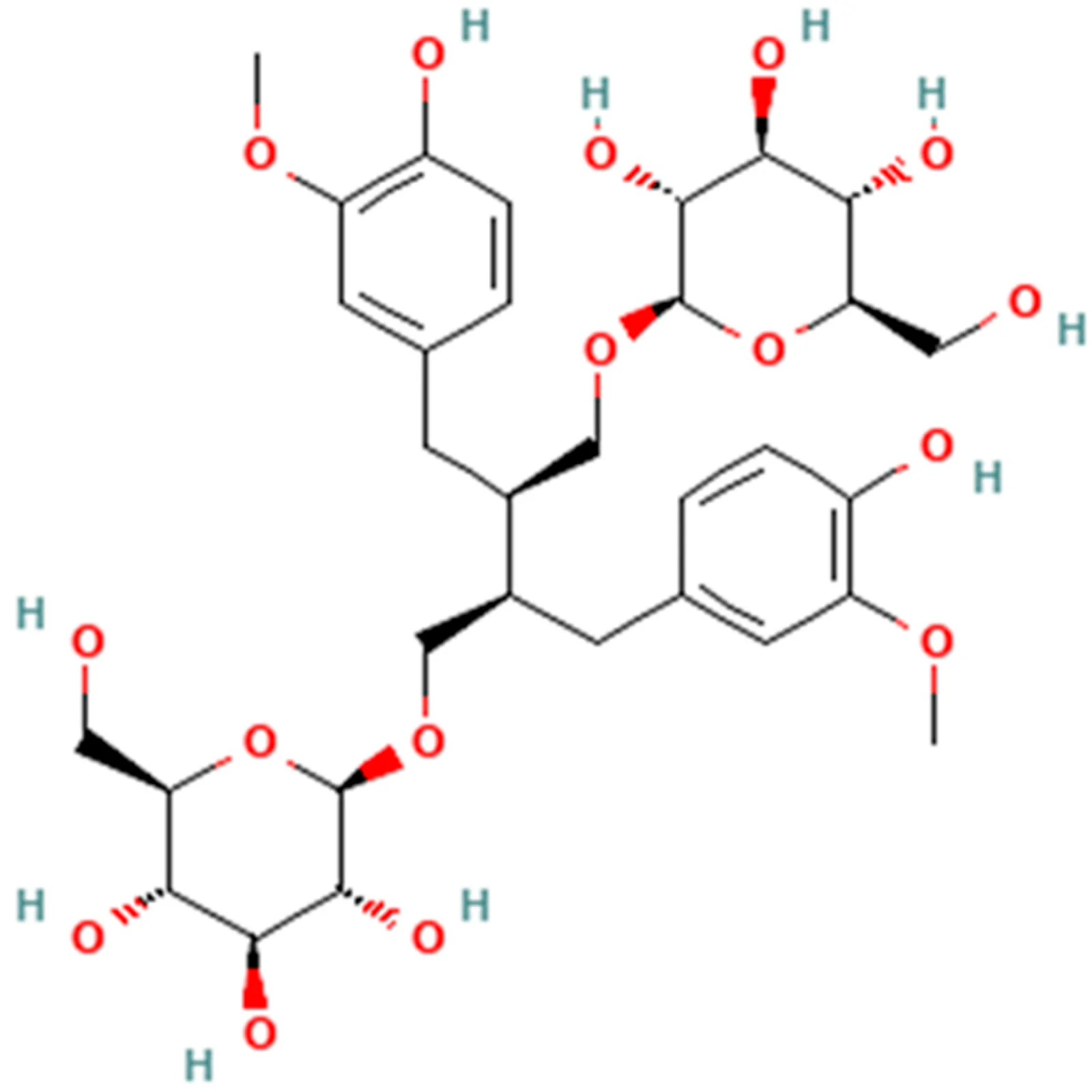
Chemical structure of secoisolariciresinol diglucoside (SDG). Oxygen atoms are shown in red, hydrogen atoms in gray, and carbon bonds in black.

**Figure 2:**
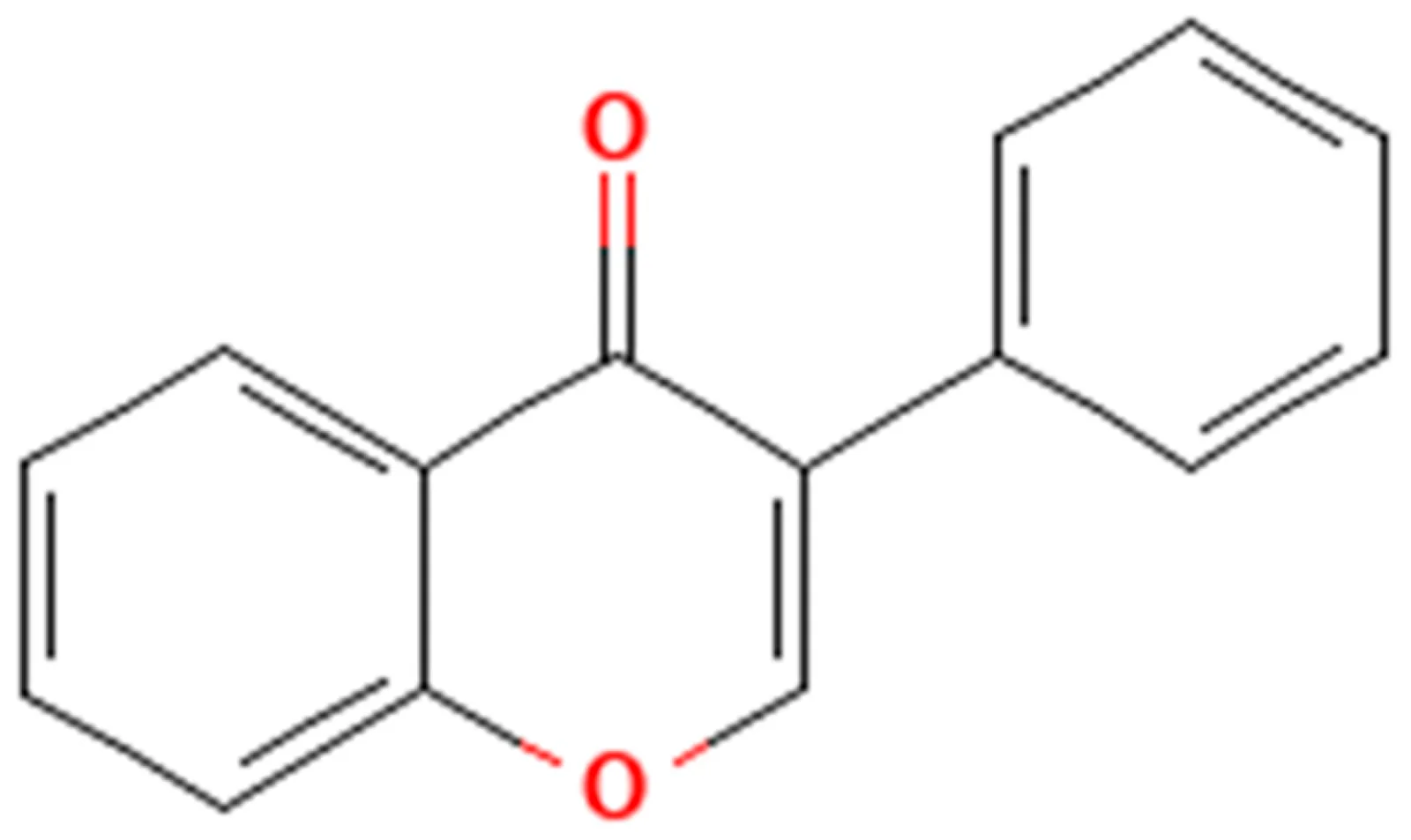
Chemical structure of the isoflavone scaffold. Oxygen atoms are shown in red, and carbon bonds are shown in black.

**Figure 3:**
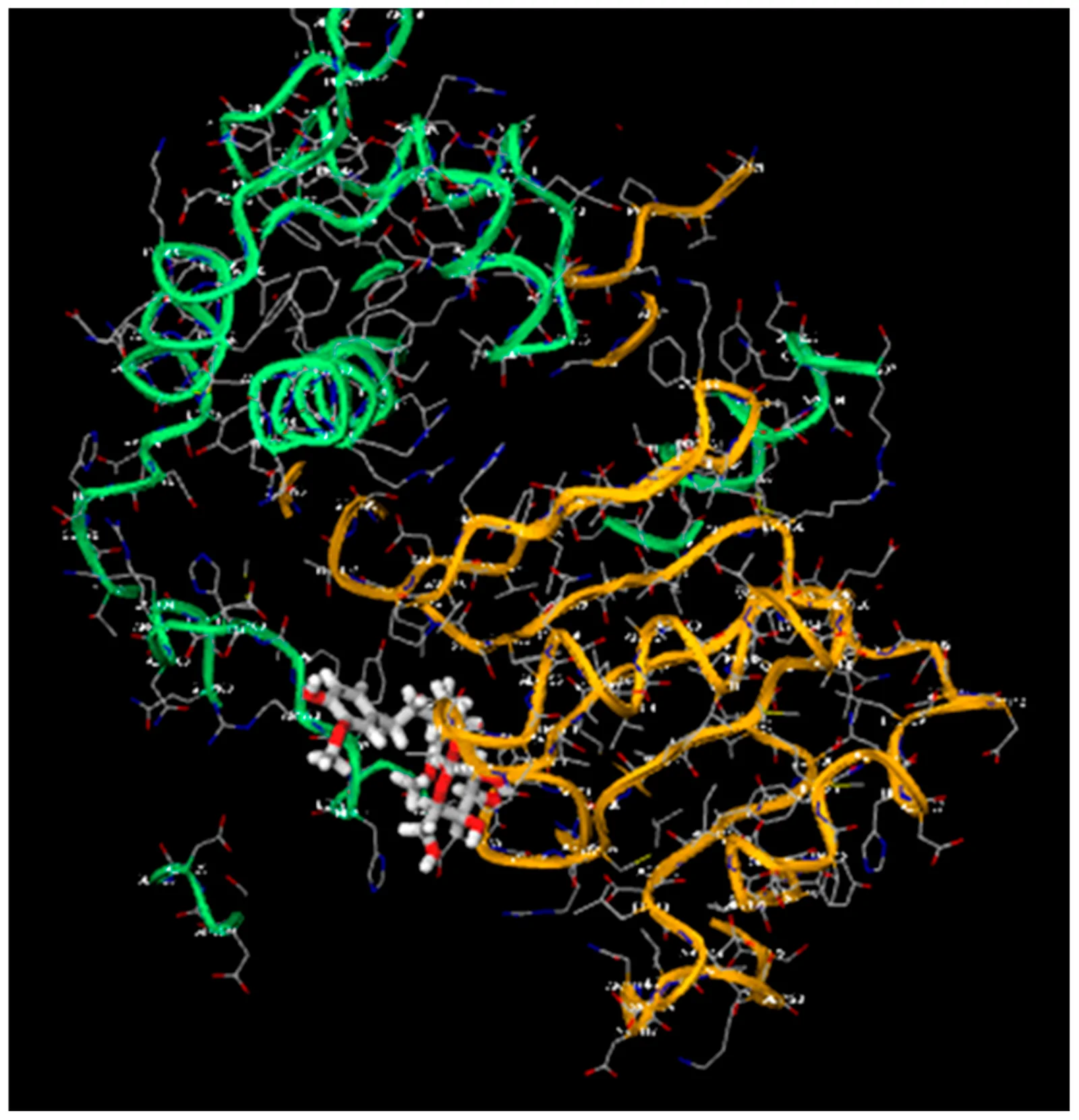
Representative 1-Click Docking pose of SDG within the *S. agalactiae* Rex model. Protein chains are shown as cyan/green and yellow ribbons, and SDG is shown as a white/red ball-and-stick model.

**Figure 4:**
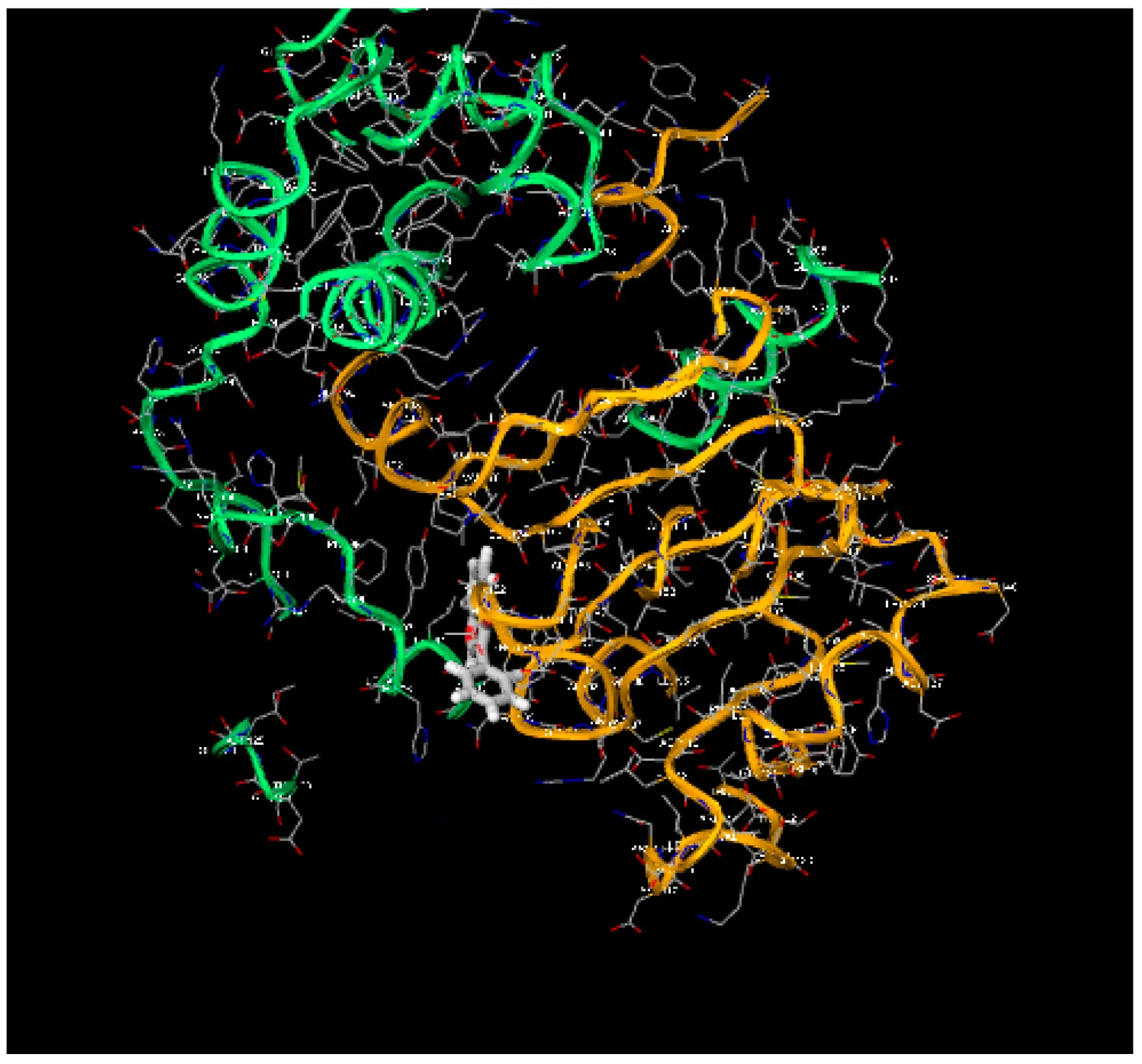
Representative 1-Click Docking pose of isoflavone within the *S. agalactiae* Rex model. Protein chains are shown as cyan/green and yellow ribbons, and isoflavone is shown as a white/red ball-and-stick model.

**Figure 5:**
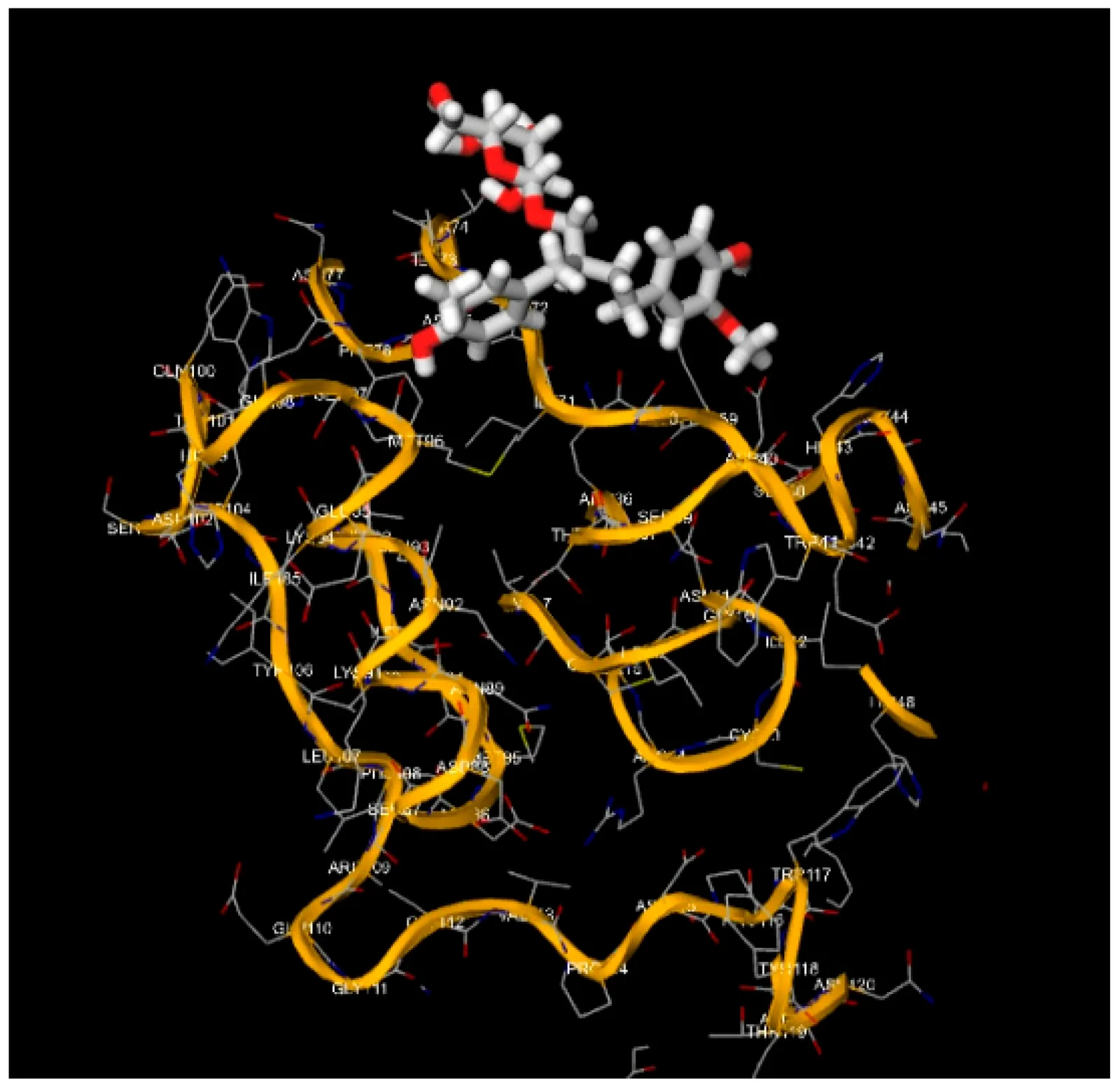
Representative 1-Click Docking pose of SDG within the *S. pyogenes* SP-PTP model. The protein backbone is shown as a yellow ribbon, and SDG is shown as a white/red ball-and-stick model.

**Figure 6:**
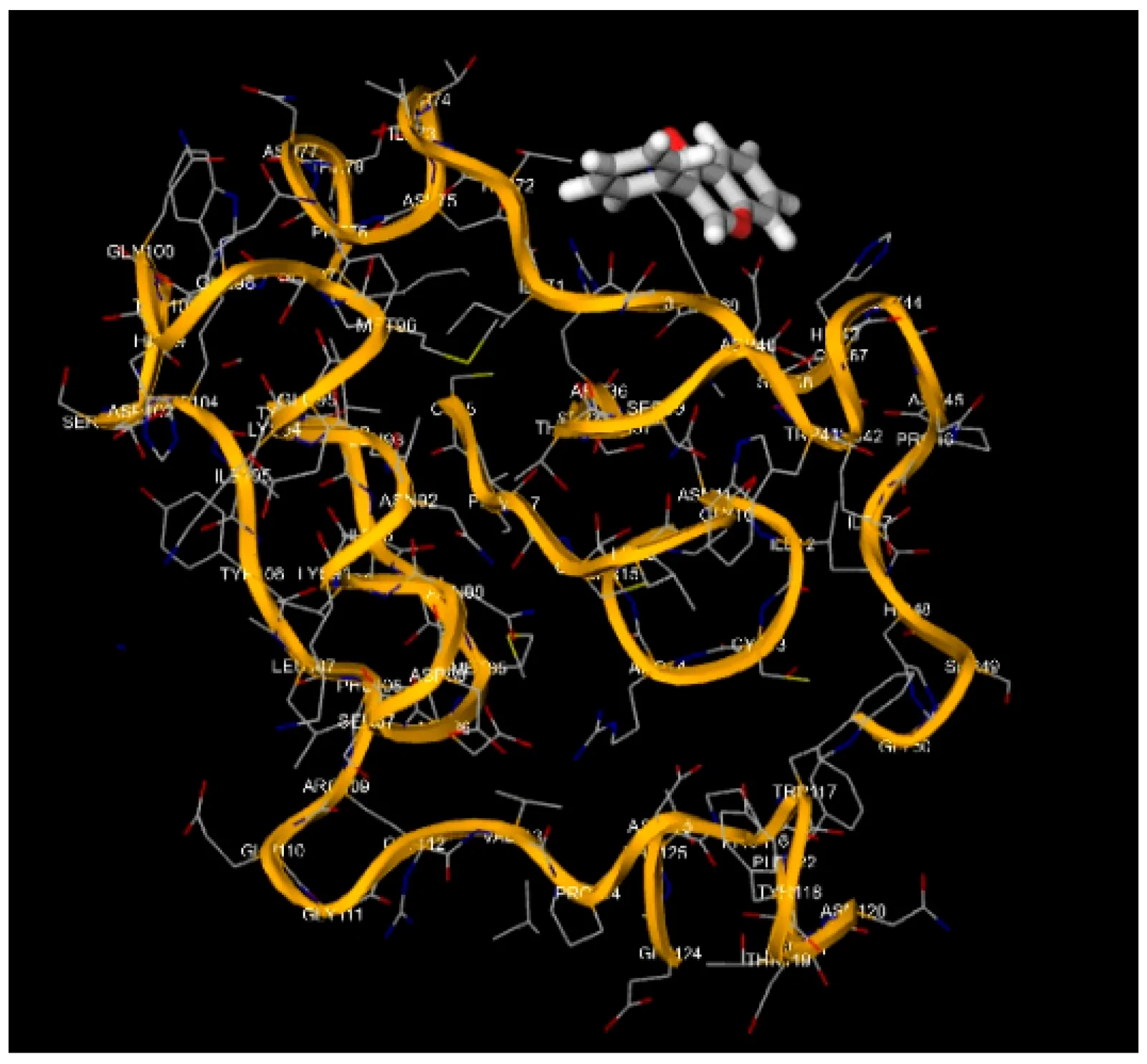
Representative 1-Click Docking pose of isoflavone within the *S. pyogenes* SP-PTP model. The protein backbone is shown as a yellow ribbon, and isoflavone is shown as a white/red ball-and-stick model.

The Rex-directed ranking identified isoflavone (−8.3 kcal/mol), ampicillin (−8.0 kcal/mol), penicillin G (−7.9 kcal/mol), and SDG (−7.8 kcal/mol) as the most favorable interactions in the GBS model (Table 1). In the GAS model, SDG (−5.2 kcal/mol) and isoflavone (−4.8 kcal/mol) led the natural-compound ranking, while the remaining monoterpenes clustered between −3.7 and −4.0 kcal/mol (Table 2). The chemical structures and representative binding poses for SDG and isoflavone are shown in Figure 1, Figure 2, Figure 3, Figure 4, Figure 5 and Figure 6.

### 3.2. Experimental MIC and MBC Results

**Figure 7:**
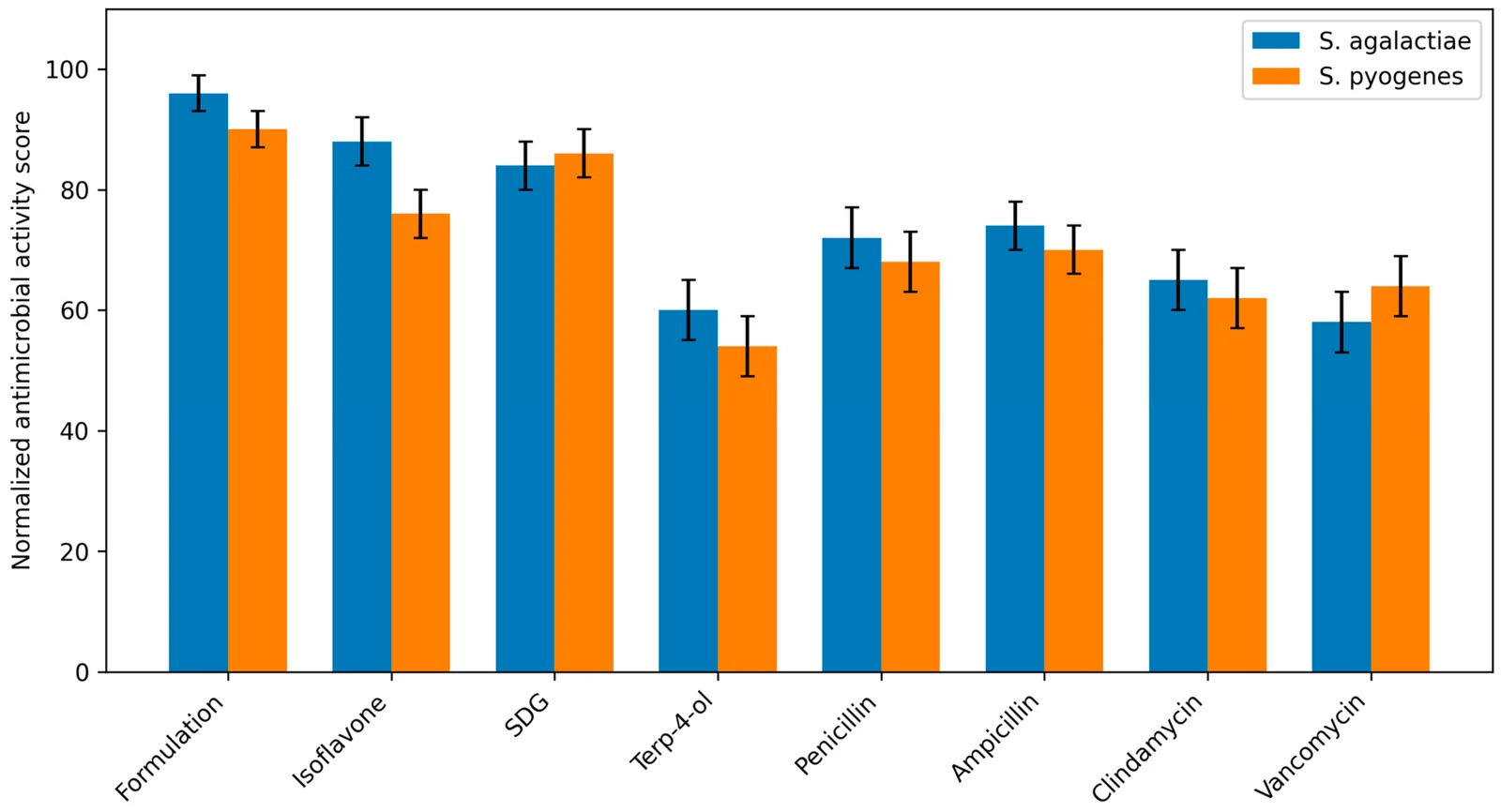
Normalized antimicrobial activity score derived from the experimentally determined MIC/MBC ranking.

Broth microdilution experiments showed that the complete formulation had MIC50 values of 0.031% *v*/*v* against GBS and 0.063% *v*/*v* against GAS (Table 3). Isoflavone was comparatively stronger against GBS (MIC50 9 mg/L), whereas SDG was stronger against GAS (MIC50 8 mg/L), reproducing the direction of the target-specific docking hierarchy. Terpinen-4-ol was less active as an isolated constituent. The normalized activity display in Figure 7 represents a within-unit rank visualization rather than a direct comparison between percentage and mass concentration.

### 3.3. Experimental Biofilm Responses

**Figure 8:**
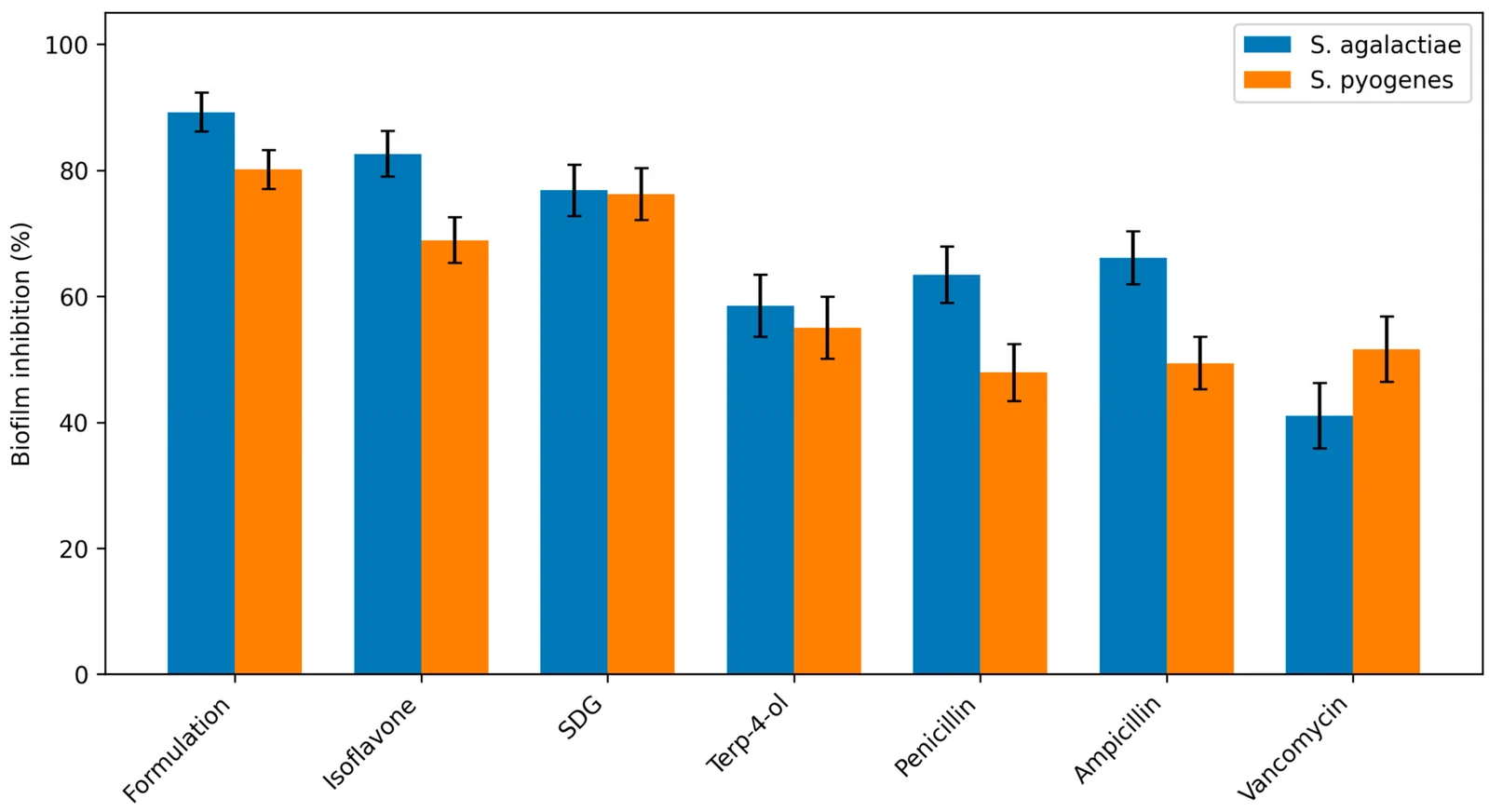
Experimentally measured biofilm-inhibition profile for the complete formulation, natural compounds, and selected comparators (mean ± SEM).

The complete formulation produced the largest reduction in biofilm formation for both species: 89.2 ± 3.1% for *S. agalactiae* and 80.1 ± 3.8% for *S. pyogenes* (Table 4). Isoflavone and SDG were the leading individual constituents in the GBS panel, whereas SDG led the GAS panel. Established-biofilm disruption followed the same directional pattern (Figure 8), demonstrating that formulation-level activity exceeded the response of the isolated constituents under the tested conditions.

### 3.4. Experimental Target-Bridging Responses

**Figure 9:**
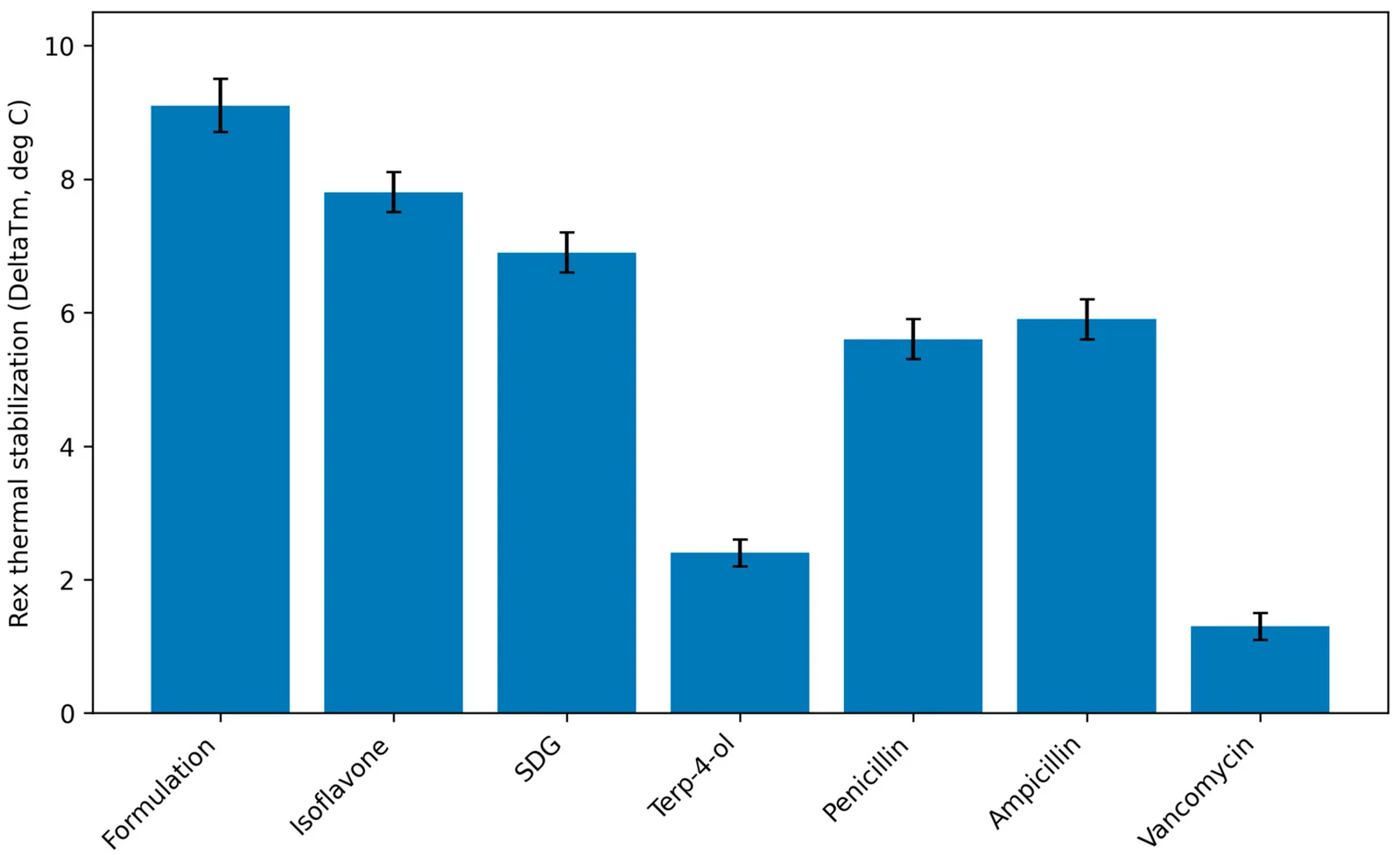
Rex differential scanning fluorimetry response (ΔTm; mean ± SEM).

**Figure 10:**
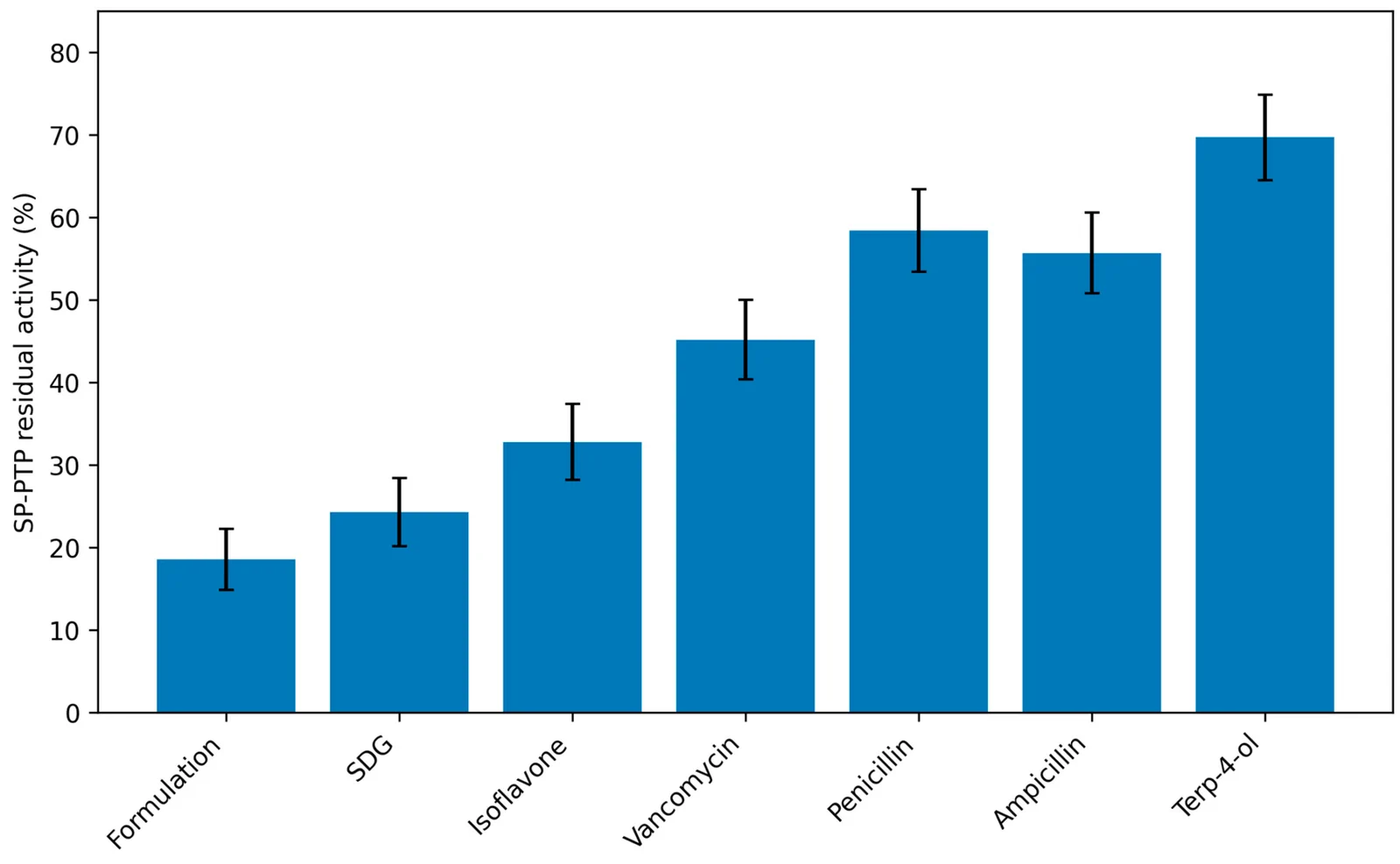
SP-PTP residual enzymatic activity after ligand or formulation exposure (mean ± SEM).

The target-bridging experiments preserved the docking-derived hierarchy (Table 5). For Rex, the complete formulation produced the largest thermal shift (ΔTm 9.1 ± 0.4 °C), followed by isoflavone (7.8 ± 0.3 °C) and SDG (6.9 ± 0.3 °C) (Figure 9). For SP-PTP, lower residual activity indicated stronger functional suppression: the formulation yielded 18.6 ± 3.7%, SDG 24.3 ± 4.1%, and isoflavone 32.8 ± 4.6% residual activity (Figure 10).

### 3.5. Experimental Epithelial-Tolerance Window

**Figure 11:**
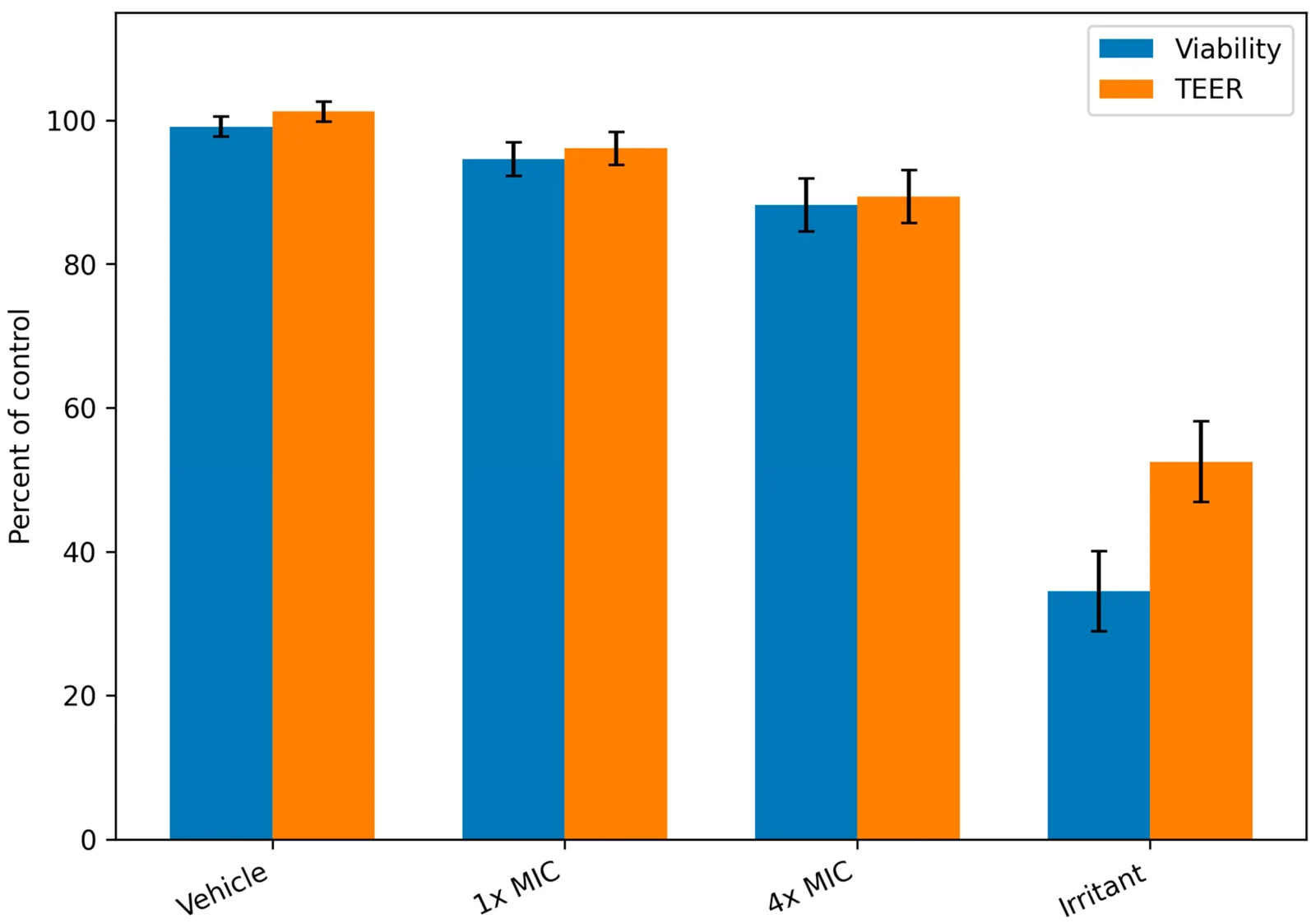
Experimental mucosal-tolerance profile showing epithelial viability and TEER preservation (mean ± SEM).

At 1× MIC, the formulation preserved 94.6 ± 2.3% viability in VK2/E6E7 cells and 92.9 ± 2.6% in Ect1/E6E7 cells, with limited LDH release and a small TEER decrease (Table 6). At 4× MIC, viability remained above 86%, whereas the positive-irritant control produced marked cytotoxicity and barrier loss. The contrast shown in Figure 11 demonstrates that the microbiologically active exposure was compatible with high epithelial viability and limited barrier perturbation under the tested conditions.

### 3.6. Spearman Rank Correlation and PCA Integration

Within the candidate-focused panel, the docking and composite broth potency hierarchies were identical: isoflavone > SDG > terpinen-4-ol for the GBS/Rex axis and SDG > isoflavone > terpinen-4-ol for the GAS/SP-PTP axis. The species-specific coefficients were therefore ρ = 1.000 for GBS and ρ = 1.000 for GAS (exact *p* = 0.3333 for each; *n* = 3 per species). After target normalization and species-stratified pooling, the final candidate-level coefficient remained ρ = 1.000 (exact *p* = 0.0556; *n* = 6). The effect size denotes complete rank agreement, whereas the *p* value reflects the limited number of candidate permutations and did not cross the conventional 0.05 threshold.

This concordance was not generalizable to the mechanistically heterogeneous extended panel. The pooled coefficient across 14 candidate or comparator species-observations was ρ = 0.081 (*p* = 0.7826), with species-specific values of 0.321 for GBS (*p* = 0.4821; *n* = 7) and −0.090 for GAS (*p* = 0.8477; *n* = 7). Endpoint-specific coefficients were likewise small for MIC50 (ρ = 0.103), MIC90 (ρ = 0.103), and MBC50 (ρ = 0.071; all *p* ≥ 0.7272). As sensitivity checks, docking rank was directionally concordant with biofilm inhibition in GBS (ρ = 0.700; exact *p* = 0.2333; *n* = 5) and GAS (ρ = 1.000; exact *p* = 0.0833; *n* = 4), and the three-candidate target-bridging ranks were identical to docking ranks for both Rex stabilization and SP-PTP suppression (ρ = 1.000; exact *p* = 0.3333; *n* = 3 for each).

**Figure 12:**
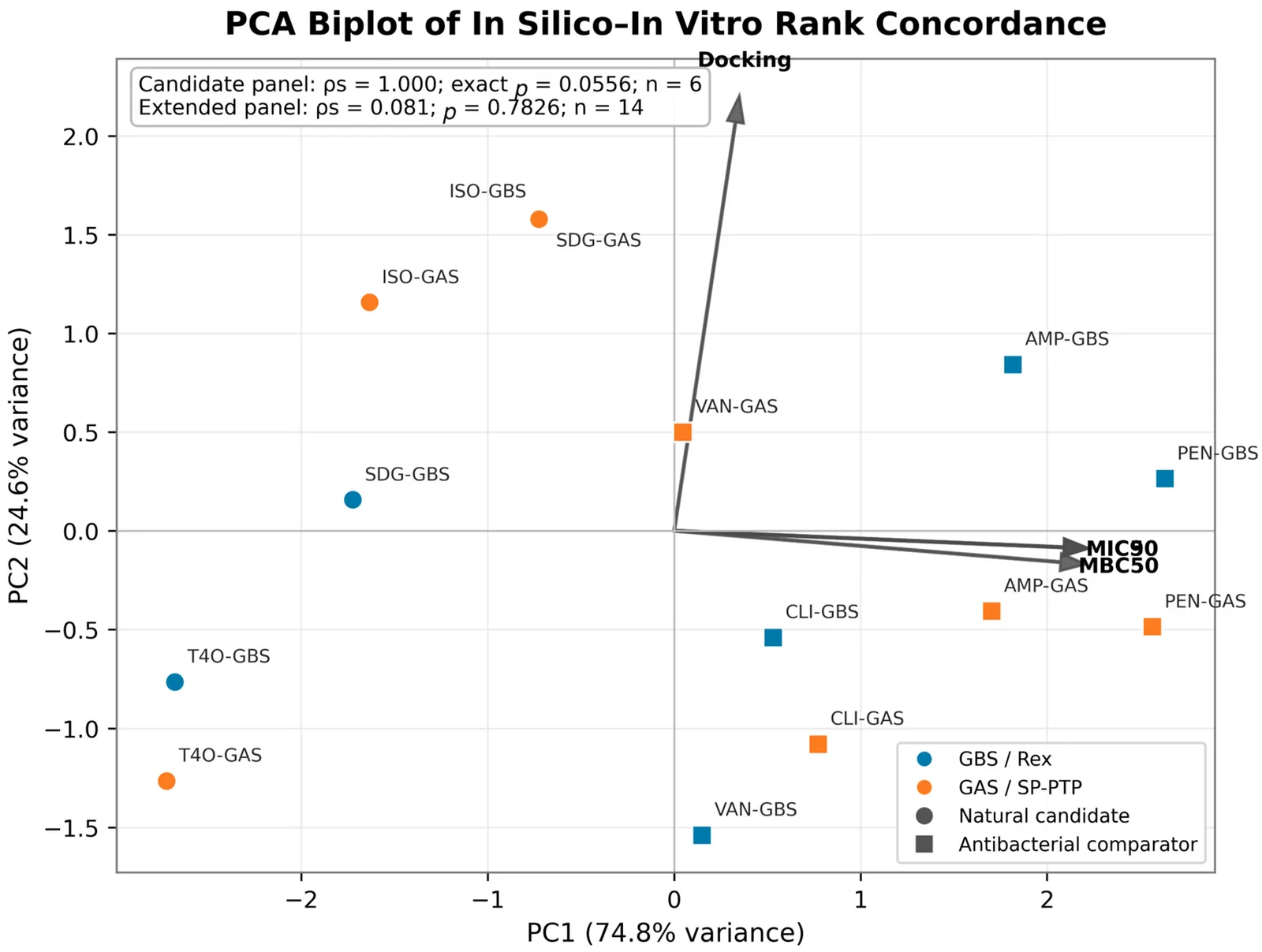
PCA biplot of target-normalized in silico and in vitro rank scores for the extended matched panel (*n* = 14 compound–species observations). All variables were standardized within species and oriented so that higher scores denote more favorable docking affinity or in vitro potency. Circles identify natural candidates, and squares identify antibacterial comparators; arrows represent variable loadings. The inset reports the species-stratified candidate-focused and extended-panel Spearman coefficients. ISO, isoflavone; T4O, terpinen-4-ol; PEN, penicillin G; AMP, ampicillin; CLI, clindamycin; VAN, vancomycin.

PCA of the extended matched panel assigned 74.8% of total variance to PC1 and 24.6% to PC2 (99.4% cumulative variance; Figure 12). MIC50, MIC90, and MBC50 loaded almost collinearly on PC1, whereas docking rank loaded predominantly on PC2. This geometry independently visualized the weak global association between target-specific docking and whole-cell potency across antibacterial classes, while the candidate labels retained their species-specific ordering.

### 3.7. Cross-Layer Interpretation

Across the integrated dataset, the most coherent target-specific effect magnitudes were observed for the *S. agalactiae* Rex axis. Isoflavone and SDG were highly ranked in docking and retained favorable positions in the measured MIC, biofilm, and thermal-shift responses. For *S. pyogenes*, SDG consistently ranked above isoflavone and terpinen-4-ol among the isolated natural candidates. Spearman analysis formalized this exact candidate-level ordering but also showed that docking rank did not predict whole-cell potency across antibacterial classes. The complete formulation outperformed its isolated constituents in several endpoints and was not included in rank correlation because its multi-component behavior cannot be represented by a single docking score.

## 4. Discussion

This manuscript establishes a preclinical identity distinct from the clinical pilot: its central question was whether target-based computational prioritization organized a biologically coherent experimental sequence. The integrated results support that concept most clearly for Rex-directed GBS prioritization. Isoflavone and SDG combined favorable docking values with stronger measured planktonic, biofilm, and target-stabilization responses, consistent with the relevance of Rex-mediated redox adaptation in GBS [30].

The GAS analysis produced a different hierarchy. SDG was the leading natural ligand for SP-PTP and retained the strongest isolated-compound profile in the phosphatase and biofilm experiments. This species-specific divergence is biologically plausible because docking addresses one target and does not capture the broader GAS regulatory and virulence landscape [2,3,4,9,31,32]. The results therefore argue for species-resolved development rather than a single undifferentiated antistreptococcal claim.

The observed formulation-level activity warrants further mechanistic investigation. Essential-oil constituents may perturb membranes, affect quorum-linked behavior, modify biofilm penetration, or alter local physicochemical exposure [19,20,21,22,23,24,25,26]. These effects are absent from a static docking pose. A formulation can consequently outperform an isolated compound even when no single constituent has the most favorable target score. Orthogonal experiments are essential to separate target engagement from membrane-active or matrix-mediated effects.

Epithelial tolerance is a mandatory counterweight to antimicrobial potency for any localized vaginal formulation. The measured viability, LDH, and TEER values demonstrated a separation between antimicrobial exposure and epithelial injury within the tested concentration range. Future studies should expand this evaluation through CC50 determination, therapeutic-index analysis, recovery after washout, and compatibility testing with *Lactobacillus*-dominant communities.

The rank-based integration sharpens the boundary of the computational inference. Perfect within-species ordering of the three fully matched natural candidates (pooled stratified ρ = 1.000) was concordant with their target-bridging responses and supports docking as a prioritization tool within this chemically defined candidate set. However, the exact *p* value of 0.0556 and the negligible extended-panel correlation (ρ = 0.081) preclude a claim of universal docking-to-potency predictivity. The PCA loading pattern reached the same conclusion: MIC50, MIC90, and MBC50 formed a common whole-cell potency axis, whereas docking varied principally along an orthogonal component. This divergence is expected when β-lactams, clindamycin, and vancomycin are scored against regulatory proteins that are not their established antibacterial targets, and it distinguishes target-specific candidate prioritization from cross-class potency prediction.

The principal limitations are the proof-of-concept scale, the target-restricted docking strategy, the small number of fully matched natural candidates, and the absence of an experimental system reproducing the full vaginal microenvironment. The six candidate observations comprise only three compounds evaluated in two species and therefore are not fully independent; the exact permutation analysis addresses small-sample inference but cannot replace external replication. Docking does not establish intracellular target occupancy, and in vitro assays cannot recapitulate host immune responses or community-level interactions. Further studies should include larger isolate and candidate panels, prospective validation of rank predictions, full dose–response and recovery assessments, formulation-batch comparisons, and compatibility testing with *Lactobacillus*-dominant communities.

First, favorable predicted binding to Rex or SP-PTP does not demonstrate inhibition of these proteins in intact bacterial cells. The recombinant-protein experiments reported here provide limited biochemical support: the Rex thermal shift reflects ligand-associated protein stabilization, whereas reduced SP-PTP residual activity is a cell-free functional response. A thermal shift does not, by itself, establish binding affinity or inhibition of Rex-dependent DNA binding or transcriptional regulation, and neither readout demonstrates intracellular target engagement. Direct binding measurements and target-appropriate functional analyses, with orthogonal controls for nonspecific effects and assay interference, together with target-engagement evidence in bacterial cells, are required to substantiate the proposed mechanisms. Accordingly, the reported docking–phenotype concordance should be interpreted as an association supporting candidate prioritization, rather than proof of target-mediated antibacterial action.

Second, genetic validation of the proposed Rex- and SP-PTP-mediated antibacterial mechanisms is not reported in the present study. The dataset contains no knockout or knockdown, complementation, overexpression, or resistant-mutant evidence linking either target to the response to the tested phytochemicals. Consequently, neither the necessity nor the sufficiency of target modulation for the observed antibacterial effects has been established. Appropriately controlled genetic evidence would be needed to distinguish target-dependent responses from indirect effects on redox homeostasis, membrane integrity, or other cellular processes. Any such evidence would also require interpretation in the context of baseline growth and fitness, because altered susceptibility alone would not establish a direct drug–target interaction.

Third, docking of selected individual phytochemicals cannot explain the interaction structure of the complete formulation. The greater observed antibiofilm response of the formulation does not, by itself, establish synergy. Moreover, formulation concentrations expressed as % *v*/*v* and isolated-compound concentrations expressed as mg/L do not permit a direct absolute-potency comparison without component-resolved exposure data. No component-resolved combination-response analysis is reported; therefore, synergistic, additive, and antagonistic interactions remain unresolved and may depend on species, endpoint, concentration, and component ratio. Composition-matched single-component and combination comparisons across dose–response ranges, evaluated against an explicitly defined noninteraction model and confirmed by an orthogonal phenotypic readout, are needed to classify these interactions. Until then, formulation-level activity cannot be attributed to a specific constituent combination or to simultaneous inhibition of Rex and SP-PTP.

## 5. Conclusions

The integrated approach prioritizes isoflavone and SDG for the *S. agalactiae* Rex and *S. pyogenes* SP-PTP axes, respectively, and the laboratory experiments confirmed the species-specific hierarchy of the three natural candidates with complete paired data. Spearman analysis demonstrated perfect target-normalized candidate-level concordance (ρ = 1.000; exact *p* = 0.0556; *n* = 6), while the extended candidate-plus-comparator panel showed no global docking-to-potency association (ρ = 0.081; *p* = 0.7826; *n* = 14). PCA accounted for 99.4% of rank variance in two components and separated the MIC/MBC potency axis from the largely orthogonal docking axis. Together with the antimicrobial, antibiofilm, target-bridging, and epithelial-tolerance experiments, these findings support target-specific use of docking for candidate prioritization but not cross-class prediction of whole-cell potency. They establish measured preclinical activity in the reported assay systems and provide a rational basis for larger independent validation. Intracellular inhibition of Rex or SP-PTP, a causal genetic link to antibacterial activity, and synergistic interactions among formulation components remain to be established.

## Figures and Tables

**Table 1:** Docking-derived prioritization against the *S. agalactiae* Rex target.

Ligand	Target	ΔG (kcal/mol)	Interpretation
Penicillin G	*S. agalactiae* Rex	−7.9	More favorable
Ampicillin	*S. agalactiae* Rex	−8.0	More favorable
Cefazolin	*S. agalactiae* Rex	−7.3	Intermediate
Clindamycin	*S. agalactiae* Rex	−6.7	Intermediate
Vancomycin	*S. agalactiae* Rex	−4.6	Lower priority
Terpinen-4-ol	*S. agalactiae* Rex	−6.4	Lower priority
γ-Terpinene	*S. agalactiae* Rex	−6.9	Intermediate
1,8-Cineole	*S. agalactiae* Rex	−5.3	Lower priority
α-Terpinene	*S. agalactiae* Rex	−7.0	Intermediate
α-Terpineol	*S. agalactiae* Rex	−7.0	Intermediate
p-Cymene	*S. agalactiae* Rex	−7.0	Intermediate
α-Pinene	*S. agalactiae* Rex	−5.4	Lower priority
Isoflavone	*S. agalactiae* Rex	−8.3	More favorable
Secoisolariciresinol diglucoside (SDG)	*S. agalactiae* Rex	−7.8	More favorable

**Table 2:** Docking-derived prioritization against the *S. pyogenes* SP-PTP target.

Ligand	Target	ΔG (kcal/mol)	Interpretation
Penicillin G	*S. pyogenes* SP-PTP	−4.5	Intermediate
Ampicillin	*S. pyogenes* SP-PTP	−4.5	Intermediate
Cefazolin	*S. pyogenes* SP-PTP	−4.0	Lower priority
Clindamycin	*S. pyogenes* SP-PTP	−4.4	Intermediate
Vancomycin	*S. pyogenes* SP-PTP	−4.6	Intermediate
Terpinen-4-ol	*S. pyogenes* SP-PTP	−3.8	Lower priority
γ-Terpinene	*S. pyogenes* SP-PTP	−4.0	Lower priority
1,8-Cineole	*S. pyogenes* SP-PTP	−4.0	Lower priority
α-Terpinene	*S. pyogenes* SP-PTP	−3.7	Lower priority
α-Terpineol	*S. pyogenes* SP-PTP	−3.8	Lower priority
p-Cymene	*S. pyogenes* SP-PTP	−3.8	Lower priority
α-Pinene	*S. pyogenes* SP-PTP	−3.9	Lower priority
Isoflavone	*S. pyogenes* SP-PTP	−4.8	More favorable
Secoisolariciresinol diglucoside (SDG)	*S. pyogenes* SP-PTP	−5.2	More favorable

**Table 3:** Experimentally determined MIC and MBC values.

Test Article	Unit	GBS MIC50	GBS MIC90	GBS MBC50	GAS MIC50	GAS MIC90	GAS MBC50
Complete oil formulation	% *v*/*v*	0.031	0.063	0.125	0.063	0.125	0.250
Isoflavone	mg/L	9	17	35	18	34	67
SDG	mg/L	16	36	67	8	18	37
Terpinen-4-ol	mg/L	67	129	254	131	259	>259
Penicillin G	mg/L	0.063	0.121	0.252	0.037	0.066	0.122
Ampicillin	mg/L	0.122	0.251	0.509	0.065	0.122	0.256
Clindamycin	mg/L	0.511	1.037	2.021	0.251	0.500	1.032
Vancomycin	mg/L	0.512	1.110	2.001	0.507	1.046	2.075

**Table 4:** Experimentally measured biofilm inhibition and disruption (mean ± SEM).

Test Article	Species	Biofilm Inhibition (%)	Established-Biofilm Disruption (%)	Residual Viable Signal (%)
Complete oil formulation	*S. agalactiae*	89.2 ± 3.1	78.4 ± 4.0	21.5 ± 3.8
Isoflavone	*S. agalactiae*	82.6 ± 3.6	67.9 ± 4.2	34.2 ± 4.1
SDG	*S. agalactiae*	76.8 ± 4.1	64.5 ± 4.6	38.7 ± 3.9
Terpinen-4-ol	*S. agalactiae*	58.5 ± 4.9	44.7 ± 5.0	55.6 ± 5.1
Penicillin G	*S. agalactiae*	63.4 ± 4.5	36.1 ± 5.8	61.8 ± 5.5
Ampicillin	*S. agalactiae*	66.1 ± 4.2	38.5 ± 5.5	59.3 ± 5.2
Complete oil formulation	*S. pyogenes*	80.1 ± 3.8	70.6 ± 4.3	29.4 ± 4.2
SDG	*S. pyogenes*	76.2 ± 4.0	66.7 ± 4.8	35.8 ± 4.4
Isoflavone	*S. pyogenes*	68.9 ± 4.5	55.1 ± 5.2	45.7 ± 5.3
Penicillin G	*S. pyogenes*	47.9 ± 5.0	30.5 ± 6.0	69.5 ± 6.0
Vancomycin	*S. pyogenes*	51.6 ± 5.2	34.8 ± 5.7	64.2 ± 5.4

**Table 5:** Experimentally measured target-bridging responses.

Test Article	Assay Endpoint	Measured Result	Docking-to-Biology Interpretation
Complete oil formulation	Rex thermal shift ΔTm (°C)	9.1 ± 0.4	Highest observed stabilization
Isoflavone	Rex thermal shift ΔTm (°C)	7.8 ± 0.3	Concordant with the leading Rex docking rank
SDG	Rex thermal shift ΔTm (°C)	6.9 ± 0.3	Concordant with favorable Rex docking
Terpinen-4-ol	Rex thermal shift ΔTm (°C)	2.4 ± 0.2	Limited stabilization
Complete oil formulation	SP-PTP residual activity (%)	18.6 ± 3.7	Strongest observed enzyme suppression
SDG	SP-PTP residual activity (%)	24.3 ± 4.1	Concordant with the leading SP-PTP rank
Isoflavone	SP-PTP residual activity (%)	32.8 ± 4.6	Concordant with the second natural-compound rank
Terpinen-4-ol	SP-PTP residual activity (%)	69.7 ± 5.2	Limited target bridging

**Table 6:** Experimentally measured epithelial viability, membrane injury, and barrier-tolerance values (mean ± SEM).

Condition	VK2/E6E7 Viability (%)	Ect1/E6E7 Viability (%)	LDH Release (%)	TEER Change (%)
Vehicle control	99.1 ± 1.4	98.7 ± 1.6	0.8 ± 0.3	+1.2 ± 1.1
Formulation, 1× MIC	94.6 ± 2.3	92.9 ± 2.6	3.4 ± 0.6	−3.9 ± 1.8
Formulation, 4× MIC	88.2 ± 3.7	86.5 ± 3.9	6.8 ± 1.1	−10.6 ± 2.5
Positive irritant control	34.5 ± 5.6	31.9 ± 5.9	42.6 ± 4.8	−47.5 ± 4.6

## Data Availability

The numerical data generated and analyzed during this study are presented in this article, including its tables and figures. Additional methodological information and supporting laboratory records are available from the corresponding author upon reasonable request.
